# Consequences of Making the Inactive Active Through Changes in Antisense Oligonucleotide Chemistries

**DOI:** 10.3389/fgene.2019.01249

**Published:** 2019-12-20

**Authors:** Khine Zaw, Kane Greer, May Thandar Aung-Htut, Chalermchai Mitrpant, Rakesh N. Veedu, Sue Fletcher, Steve D. Wilton

**Affiliations:** ^1^ Centre for Molecular Medicine and Innovative Therapeutics, Murdoch University, Perth, WA, Australia; ^2^ Department of Biochemistry, Faculty of Medicine Siriraj Hospital, Mahidol University, Bangkok, Thailand; ^3^ Perron Institute for Neurological and Translational Science and Centre for Neuromuscular and Neurological Disorders, The University of Western Australia, Perth, WA, Australia

**Keywords:** *DMD*, antisense oligonucleotide, locked nucleic acid, locked nucleic acid/2′-*O*-methyl mixmer, cryptic splice site

## Abstract

Antisense oligonucleotides are short, single-stranded nucleic acid analogues that can interfere with pre-messenger RNA (pre-mRNA) processing and induce excision of a targeted exon from the mature transcript. When developing a panel of antisense oligonucleotides to skip every dystrophin exon, we found great variation in splice switching efficiencies, with some antisense oligonucleotides ineffective, even when directed to canonical splice sites and transfected into cells at high concentrations. In this study, we re-evaluated some of these ineffective antisense oligonucleotide sequences after incorporation of locked nucleic acid residues to increase annealing potential. Antisense oligonucleotides targeting exons 16, 23, and 51 of human *DMD* transcripts were synthesized as two different chemistries, 2′-*O*-methyl modified bases on a phosphorothioate backbone or mixmers containing several locked nucleic acid residues, which were then transfected into primary human myotubes, and *DMD* transcripts were analyzed for exon skipping. The ineffective 2′-*O*-methyl modified antisense oligonucleotides induced no detectable exon skipping, while all corresponding mixmers did induce excision of the targeted exons. Interestingly, the mixmer targeting exon 51 induced two unexpected transcripts arising from partial skipping of exon 51 with retention of 95 or 188 bases from the 5′ region of exon 51. These results indicated that locked nucleic acid/2′-*O*-methyl mixmers are more effective at inducing exon skipping, however, this improvement may come at the cost of activating alternative cryptic splice sites and off-target effects on gene expression.

## Introduction

Mutations in the *DMD* gene are responsible for Duchenne (DMD) and Becker (BMD) muscular dystrophies. The more severe DMD is typically associated with frameshifting deletions, duplications, or insertions, or nonsense mutations that cause disruption of the open reading frame (ORF). Most mutations that do not disrupt the ORF produce an internally truncated but partially functional protein, resulting in the milder BMD phenotype (Monaco et al., 1988). This spectrum of disease severity underlies the development of therapeutic interventions such as antisense oligonucleotide (AO) induced targeted exon skipping to treat DMD (Fletcher et al., 2017; Stein and Castanotto, 2017).

AOs are short, single-stranded nucleic acid analogues that are designed to anneal to a messenger RNA (mRNA) or pre-mRNA through Watson–Crick base pairing interactions and, depending on the base and backbone chemistries, induce a variety of mechanisms to alter the gene expression. AO induced splice-switching strategies to skip one or more specific exons, with restoration of the ORF and expression of dystrophin with improved function has been explored as a treatment for DMD. *Exondys 51* (Sarepta Therapeutics), a phosphorodiamidate morpholino oligomer targeting exon 51, has been given accelerated approval by the US Food and Drug Administration (FDA) for the treatment of DMD (Syed, 2016).

**Figure 1:**
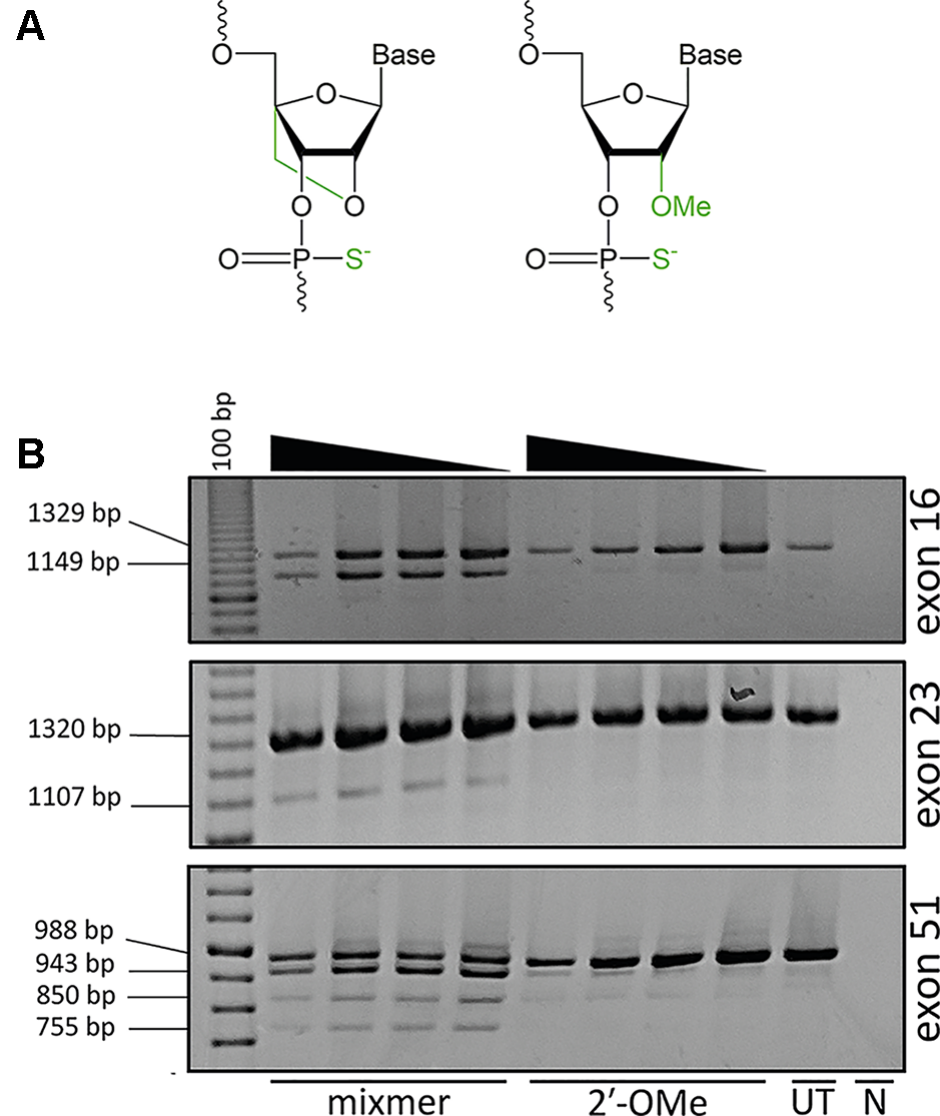
Analysis of exon skipping efficiency using LNA/2′-OMe (locked nucleic acid/2′-*O*-methyl) mixmers and 2′-OMe modified antisense oligonucleotides (AOs). **(A)** Structure of LNA; 2′ oxygen and 4′ carbon of the sugar ring is connected by an extra bridge (left) and 2′-OMe; a methyl group is added to the 2′ hydroxyl of the sugar ring (right). The backbone is modified with a phosphorothioate linkage where the non-bridging oxygen is replaced with a sulfur. **(B)** Reverse transcriptase polymerase chain reaction (RT-PCR) analysis of RNA extracted from primary human myotubes cultures transfected with LNA/2′-OMe mixmers or 2′-OMe. All the mixmers induced skipping of the targeted exons, while 2′-OMe AOs showed no exon skipping. The mixmer targeting exon 51 produced two RT-PCR amplicons in addition to the expected full length and exon 51–skipped transcripts. The arrowheads indicate decreasing AO concentration (200, 100, 50, and 25 nM). (UT: untreated cells, N: no template negative RT-PCR control, 100bp: DNA ladder).

Strategies to improve AO potency through more efficient cellular uptake or increased stability and specificity of AOs are continually being explored to develop compounds that would confer optimal therapeutic effects. Approaches to enhance AO potency involve chemical modifications of the phosphate backbone or at the 2′ position of the ribose sugar, such as 2′-*O*-methyl (2′-OMe) or locked nucleic acid (LNA) in order to increase binding affinity and resistance against nuclease degradation (**Figure 1A**). LNA-modified oligonucleotides show a high binding affinity and increased stability against nuclease degradation compared to only the 2′-OMe modification (Kierzek et al., 2009).

When developing a panel of AOs to skip all dystrophin exons, remarkably we found that two out of three compounds could induce some level of exon skipping, albeit at variable efficiencies. During development of splice switching AOs, targeting many of the canonical donor or acceptor splice sites in the dystrophin primary transcript appeared to be largely ineffective, especially when compared to targeting intra-exonic splice enhancer motifs (Adams et al., 2007). The current study aimed to ascertain if increasing the annealing potential of an AO by incorporating LNA residues at selected positions could influence splicing. We selected three examples of acceptor or donor splice sites that were ineffective as 2′-OMe AO target sites. We found that the LNA/2′-OMe AO mixmers with increased annealing potential were capable of modifying pre-mRNA processing, indicating that these AOs could act as splice switching compounds if the strength of annealing was sufficiently increased. However, in one case, multiple transcripts were induced due to the activation of intra-exonic cryptic splice sites, suggesting that enhanced annealing may compromise splice switching specificity.

## Materials And Methods

### Design and Synthesis of Chemically Modified AOs

2′-OMe AOs, previously designed, evaluated, and found to be inactive with respect to inducing skipping of human dystrophin exons 16, 23, and 51 (Harding et al., 2007; Mitrpant et al., 2009), were resynthesized in house on a GE AKTA Oligopilot plus 10 (GE Healthcare Life Sciences) oligonucleotide synthesizer, as described previously using the 1 μmol thioate protocol (Le et al., 2017). The mixmers incorporating the LNAs are described in **Table 1** and were also synthesized in house (Le et al., 2017).

### Cell Culture and Transfection

Primary human myoblasts, obtained after informed consent and approved by the Murdoch University human ethics committee (#2013_156), were cultured and differentiated into myotubes as described by Rando and Blau (Rando and Blau, 1994) with minor modifications (Harding et al., 2007). Briefly, myoblasts were seeded on 24-well plates coated with poly-D-lysine (Merck Millipore), and Matrigel (Corning, supplied through In Vitro Technologies) at a density of 30,000 cells/well. Cells were differentiated into myotubes in 5% horse serum low glucose Dulbecco's modified Eagle medium (DMEM) (Thermo Fisher Scientific) by incubating at 37°C in 5% CO_2_ for 48 h. All 2′-OMe AOs and mixmers were transfected into differentiated myotubes in Opti-MEM (Invitrogen) as cationic lipoplexes with Lipofectamine 2000 reagent at 1:1 w:w ratio, according to the manufacturer’s instructions (Invitrogen) in a final transfection volume of 500 µl/well in a 24-well plate. Transfected cells were incubated for 48 h before total RNA extraction.

### RNA Extraction and RT-PCR

RNA extraction was carried out using the MagMAX-96 Total RNA Isolation Kit (Life Technologies), according to the manufacturer’s instructions. RT-PCRs were performed using the One-Step SuperScript III RT-PCR kit (Life Technologies) as per manufacturer’s instructions. The temperature profile was 55°C for 30 min, 94°C for 2 min, followed by 35 cycles of 94°C for 15 s, 55°C for 30 s, and 68°C for 1 min 10 s. RT-PCR products were separated on 2% agarose gels in Tris-acetate-ethylenediaminetetraacetic acid (EDTA) buffer, and the images were captured on a Fusion FX gel documentation system (Vilber Lourmat, Marne-la-Vallee, France).

### Band-Stab PCR and Sequencing

Individual bands representing RT-PCR amplicons were purified and amplified by band-stab PCR (Wilton et al., 1997) using AmpliTaq Gold DNA Polymerases (Thermo Fisher Scientific) with the thermal profile of 94°C for 5 min followed by 25 cycles of 94°C for 15 s, 50°C for 15 s, 72°C for 1 min with the final extension of 72°C for 5 min. Amplicon sequences were identified by Sanger sequencing at the Australian Genome Research Facility (AGRF, Perth, Australia). The nucleotide sequences were deposited at GenBank and available as accession numbers MN490082–MN490085.

### *In Silico* Analysis

The natural and potential donor splice sites of *DMD* exon 51 were analyzed using Human Splicing Finder 3.1 (http://www.umd.be/HSF3/HSF.shtml) (Desmet et al., 2009).

## Results

### AO Synthesis and Transfection

In this study, we used AO sequences that had been designed to induce skipping of exons 16, 23, and 51 from the human dystrophin gene transcript, but were previously reported to be largely ineffective when transfected into cells as 2′-OMe modified compounds on a phosphorothioate backbone. These sequences were re-synthesized as 2′-OMe AOs and as respective LNA-2′-OMe “mixmers” on a phosphorothioate backbone, a 20-mer for exon 16 and 25-mers for exons 23 and 51, as shown in **Table 1**. Primary human myoblasts obtained after informed consent and approved by the Murdoch University human ethics committee (#2013_156) were cultured on 24-well plates, differentiated over 48 h, and transfected with AO cationic lipoplexes at 200, 100, 50, and 25 nM concentrations. The cells transfected with either 2′-OMe or LNA/2′-OMe mixmer cationic lipoplexes were healthy and showed no obvious signs of toxicity or cell death at these concentrations.

**Table 1:** Sequences of AOs used in this study.

Sequence	LNA/2′-OMe Mixmers	2′-OMe
H16A (–07+13)	C**C**G**C**U**U**U**U**A**A**AACCUG**U**U**A**A	CCGCUUUUAAAACCUGUUAA
H23D (+07–18)	AG**U**A**A**AA**U**C**U**UGAA**U**UA**C**C**U**GAA**U**U	AGUAAAAUCUUGAAUUACCUGAAUU
H51D (+07–18)	UA**U**CA**U**U**U**UUUCUCA**U**ACC**U**UC**U**G**C**	UAUCAUUUUUUCUCAUACCUUCUGC

LNA nucleotide monomers are represented as bold characters.

### Evaluation of AO Efficiency

The cells were collected 48 h after transfection and analyzed for respective *DMD* exon skipping by reverse transcriptase polymerase chain reaction (RT-PCR). As anticipated, the 2′-OMe AOs did not induce exon skipping while all the LNA/2′-OMe mixmers induced consistent skipping of the targeted exons at all tested concentrations (**Figure 1B**). Interestingly, the LNA/2′-OMe mixmer targeting exon 51 induced two transcript products in additional to the predicted full-length products (988 bp) and exon 51–skipped (755 bp) transcript product. The 2′-OMe AO directed at dystrophin exon 51 donor splice site induced very weak exon skipping and also low levels of the 943 and 850 bp amplicons.

### Identification of Full-Length and Skipped Transcripts

**Figure 2:**
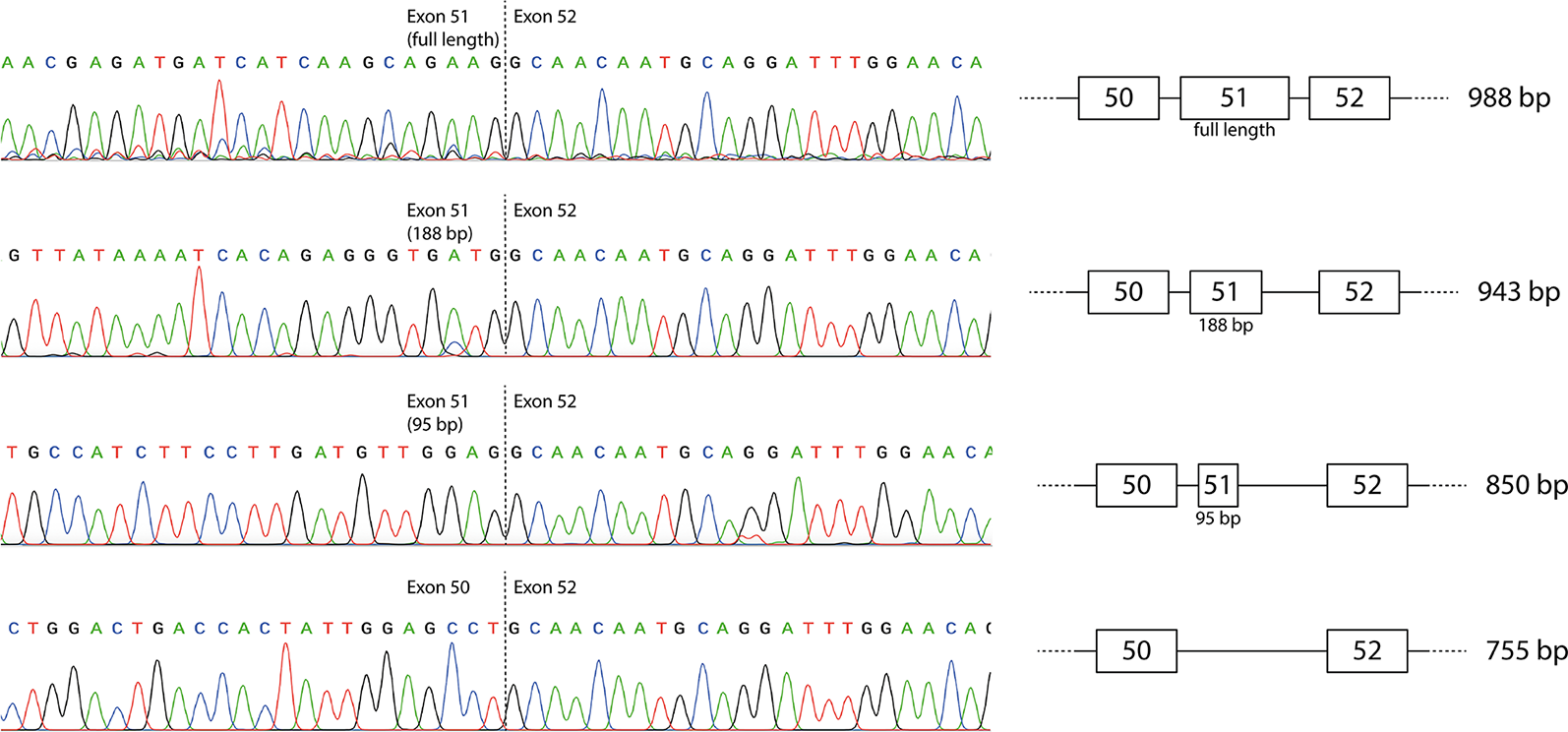
Sequencing of individual reverse transcriptase polymerase chain reaction (RT-PCR) products produced by the mixmer targeting exon 51. The two additional amplicons represent incomplete skipping of exon 51, with retention of the first 95 or 188 bases of exon 51.

The individual products were purified by band-stab PCR (Wilton et al., 1997) and identified by direct DNA sequencing that confirmed the identity of the full-length and induced exon-skipped amplicons (**Figure 2**). The additional products generated predominantly by the exon 51 LNA/2′-OMe mixmer arose from activation of cryptic donor splice sites, with retention of 95 or 188 bases from the beginning of exon 51 (850 and 943 bp amplicons respectively). The sequences are available at GenBank with the accession numbers MN490082–MN490085.

### Analysis of Activated Cryptic Splice Sites

**Figure 3:**
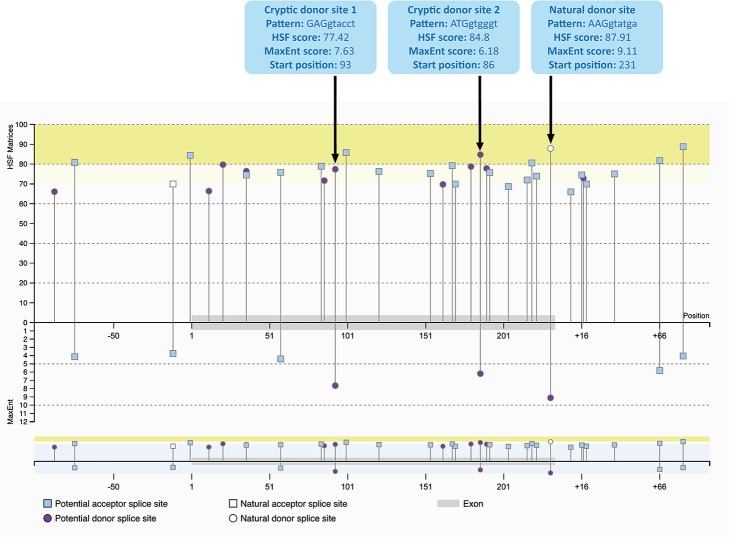
Potential splice sites for human *DMD* exon 51 predicted by Human Splicing Finder 3.1 (HSF) indicating the natural donor splice site and the two cryptic donor splice sites activated by the mixmers. The splice sites scores are predicted for each site (Yeo and Burge, 2004; Desmet et al., 2009). The splice site score for the cryptic donor site 2 is higher than the cryptic donor site 1 in HSF score, although the cryptic donor site 1 has a higher score in the maximum entropy model.

The cryptic splice sites activated by the mixmer targeting exon 51 are potential splice sites within *DMD* exon 51 (**Figure 3**) as predicted by Human Splicing Finder 3.1 (HSF) (http://www.umd.be/HSF3/HSF.shtml) (Desmet et al., 2009). Both cryptic donor splice sites included the canonical donor splice site sequence, ‘GT’ at the 5′ end of the “intron.” The HSF scores for cryptic donor sites 1 and 2 are 77.42 and 84.8 respectively, which are high and close to the natural splice site score, 87.91. In the maximum entropy model, both cryptic sites are also the only positions predicted to be potential splice sites in addition to the natural donor site (Yeo and Burge, 2004). Interestingly, the splice site score for the cryptic donor site 2 is higher than the cryptic donor site 1 in HSF score, although the cryptic donor site 1 has a higher score in the maximum entropy model.

## Discussion

The most common consequence of mutations of the canonical donor or acceptor splice sites is exon skipping. However, the majority of dystrophin exons appeared unresponsive to splice switching AOs targeting these motifs, and only 2 exons out of the 77 were identified with the donor splice sites being optimal targets (Wilton et al., 2007). In this study, we modified three previously reported inactive AO sequences identified by ourselves and others, with LNA incorporation to improve the annealing affinity, and revisited their ability to induce exon skipping. Consistent with the previous results, newly synthesized 2′-OMe AOs showed no or poor exon skipping after transfection, while all mixmers induced readily detectable skipping of the targeted exons.

During optimization of AOs to excise *DMD* exon 16, we previously demonstrated that AOs targeting the acceptor site and adjacent putative exonic splicing enhance sites could induce marked exon skipping, as long as the AOs were 25-mers or longer (Harding et al., 2007). While overlapping 25-mer and 31-mers induced robust exon 16 skipping, a shorter 20-mer common to all sequences was ineffective. Since shorter AOs are more efficiently and economically synthesized, we aim to identify the shortest sequence capable of inducing robust exon skipping, but in this instance, the 20-mer did not have sufficient annealing strength to the target because of the low ‘GC’ content (Harding et al., 2007). Incorporation of LNAs into this short and inactive 20-mer resulted in increased annealing potential and relatively robust exon skipping. This suggested that the limitation of the original 20-mer was due to weak hybridization.

A similar trend was observed for AO induced human dystrophin exon 23 skipping. We previously reported refining 2′-OMe AOs designed to induce exon 23 skipping and restore dystrophin expression in the *mdx* mouse (Mann et al., 2002). In subsequent studies, we found that the optimal annealing coordinates to skip mouse dystrophin exon 23, H23D (+07–18), were not an effective target for excising human dystrophin exon 23 (Mitrpant et al., 2009). In this study, we synthesized the same sequence as a LNA/2′-OMe mixmer, and exon skipping was observed when AO was applied to cultured human myoblasts. Nevertheless, this increased annealing potential only resulted in modest levels of exon skipping and would not be considered for clinical application.

Dystrophin exon 51 was selected as the exon target for the first *DMD* exon skipping clinical trials since excising this exon would be relevant to the largest subset of DMD patients (Aartsma-Rus et al., 2009; Bladen et al., 2015). During the pre-clinical development of *Eteplirsen,* all the oligomers targeting the donor splice site of human dystrophin exon 51were found to be largely ineffective (Arechavala-Gomeza et al., 2007). A 25-mer 2′-OMe AO, H51D(+08–17), failed to skip exon 51, whereas another AO, a 23-mer with higher GC content, H51D(+16–07), induced only weak exon skipping (Harding et al., 2007). We resynthesized the AO sequence H51D (+07–18) as a mixmer and showed induction of three different transcripts: complete skipping of exon 51 together with two other transcripts produced by the activation of intra-exonic cryptic donor sites. The levels of complete exon 51 skipping were modest, and thus, such an approach would not be clinically applicable to restore the reading frame. One of the few examples of activation of cryptic splice sites in the dystrophin gene transcript was in exon 53 of the mouse dystrophin gene, 77 bases upstream of the normal donor splice site (Mitrpant et al., 2009). We anticipated that cryptic splice site activation would be far more common than we have actually encountered to date.

Our results show that the incorporation of LNA bases into the AOs increased the annealing potential and transformed inactive antisense sequences into active AOs. However, one significant concern that must be investigated is the extent to which cryptic splice sites are activated, leading to partial exon skipping. LNA-fully-modified AOs containing up to three mismatches induced dystrophin exon 46 skipping in DMD patient cells, indicating low sequence specificity, with the possibility of off-target binding potential (Aartsma-Rus et al., 2004). In addition, gapmers modified with LNAs show acute hepatotoxicity in mice (Kasuya et al., 2016). We and others have previously reported that AOs on a fully modified phosphorothioate backbone recruited nuclear proteins involved in RNA processing and induced global disturbance of cellular processes (Shen et al., 2014; Flynn et al., 2018).

In conclusion, we show that the incorporation of LNAs into 2′-OMe antisense sequences increased their potency as steric blockers of splicing, thereby making the inactive active. However, this enhancement came at a cost in efficiency and specificity due to activation of cryptic splicing, raising the risk of adverse and off-target effects elsewhere in the human transcriptome.

## Data Availability Statement

The nucleotide sequences were deposited at GenBank and available as accession number MN490082-MN490085.

## Author Contributions

Conceptualization, SW, SF, and RV. Experiments, KZ and KG. Writing—original draft preparation, KZ. Writing—review and editing, KZ, MA-H, SW, and SF. Supervision, MA-H, CM, SW, and SF.

## Funding

This work is supported by the National Health and Medical Research Council (Australia) grant APP1144791.

## Conflict of Interest

SW, KG, and SF are named inventors on exon skipping patents and as such are entitled to royalty and milestone payments as they arise.

The remaining authors declare that the research was conducted in the absence of any commercial or financial relationships that could be construed as a potential conflict of interest.
